# Public good experiment data of a water game framed to Rajasthan/India

**DOI:** 10.1016/j.dib.2019.104556

**Published:** 2019-09-24

**Authors:** Thomas Falk, Shalander Kumar, Srinivasa Srigiri

**Affiliations:** a Research Program on Innovation Systems for the Drylands, International Crops Research Institute for the Semi-Arid Tropics, Patancheru, 502 324, Telangana, India; b Social Outlook Consulting, Plot 636 and 629, Matrusri Nagar, Miyapur, Hyderabad, 500 049, Telangana, India

**Keywords:** Economic field experiment, Capacity development, Infrastructure maintenance, Communication, Collective action

## Abstract

This dataset belongs to a framed economic field experiment conducted in 2016 in Bhilwara district in Rajasthan state in India. A public good game was framed as dam management challenge. We made incentivized payments based on the game earnings. The data are organized as a panel defined by players and experiment rounds. The dataset contains the experiment decisions in different phases of the experiment as well as socio-economic variables of the anonymized players. The data can be accessed through the Dataverse of the International Crops Research Institute for the Semi-Arid Tropics (ICRISAT) under the following link: https://doi.org/10.21421/D2/MFT8ZD. The data article is related to the research article Falk et al. [2] on “Experimental games for developing institutional capacity to manage common water infrastructure in India”.

**Table:** Specifications Table

Subject area	Economics
More specific subject area	Behavioral Economics
Type of data	Table uploaded at ICRISAT Dataverse
How data was acquired	Economic field experiments conducted with farmers in Rajasthan/India
Data format	cleaned and labeled raw data
Experimental factors	Three stage game: first phase with discrete decisions; second phase providing social information and permitting communication; third stage with game variation slightly changing the game structure.
Experimental features	The field experiment was framed as dam management challenge. Participants had to invest a share of an endowment in the maintenance of the dam. A non-linear pay-off function similar to the one in the irrigation games of Cardenas et al. [1] and Janssen et al. [4], [5] was used. The accumulated individual contributions determine the group earning. The group earning was distributed equally among the players. We made incentivized payments based on the total game earnings.
Data source location	30 randomly sampled communities in Bhilwara district in Rajasthan state in India; Data were collected by the International Crops Research Institute for the Semi-Arid Tropics (ICRISAT)
Data accessibility	The data can be accessed through the ICRISAT Dataverse: https://doi.org/10.21421/D2/MFT8ZD
Related research article	Falk, T., Kumar, S., Srigiri, S., 2019. Experimental games for developing institutional capacity to manage common water infrastructure in India, Agricultural Water Management. 221: 260–269 [2].

**Table:** 

**Value of the data** • Especially the first experiment phase used a standard frame of irrigation games based on the design of Cardenas et al. [1] and Janssen et al. [4], [5]. Multiple studies have used a similar design. Hence, experimental economists and social scientists can use the data e.g. for intercultural comparisons similar to Henrich [3]. • Experimental economists and social scientists could further use the data for studies on various game features. Diverse elements of the design such as the framing, the pay-off function, or the payment scheme could be changed as new treatments to test methodological hypothesis. • The same experiment could be conducted in a laboratory setting e.g. with students in order to compare behavioral patterns of the two groups. • The data can be used for synthesis analyses of public good games. It can be used to compare different types of public good games in order to test hypothesis related to the social-ecological context. • Especially the first experiment phase is expected to reveal internalized behavioral patterns and social preferences. Field researchers can use the data as behavioral context information for diverse future studies.

## 1 Data

The data stem from a framed public good experiment replicating challenges associated with minor irrigation infrastructure management. Research was carried out in Bhilwara district in Rajasthan state in India.

The experiment design has the general features of a standard irrigation game as presented by Cardenas et al. [1] and Janssen et al. [4], [5]. Details on the design are provided in the subsequent section Experimental Design, Materials, and Methods.

The resulting data are organized as a panel defined by player IDs (variable player id) and experiment rounds (variable round). Each player conducted multiple rounds and made in each round an investment decision (variable investment). Based on the pay-off function the player received an earning from the public good (variable dam_earn) to which the saved endowment was added to calculate the total round earning (variable round_earn).

The experiment was conducted in 30 communities (variable site_ID) with 10 players each. It was played in three phases: first phase with discrete decisions (variable Variation – value baseline); second phase providing social information and permitting communication (variable Variation – value communication); third stage with game variation slightly changing the game structure (variable Variation – all other values). The data include basic socio-economic variables and counts of players' contributions to discussions during the game.

The data are made available in csv and Stata data formats. The STATA data file has the advantages that it contains more detailed variable and value labels. In addition, a file describing the data is available under ICRISAT Dataverse.

## 2 Experimental design, materials, and methods

Like all public good experiments, the game focused on the provisioning action situation, namely the maintenance of the common community water infrastructure. Pay-offs were adjusted to region-specific estimates of average maintenance costs of dams as well as typical income derived from dam management.

At the beginning of each game round, players received an initial endowment of 5000 Play Rupees. In the first five game rounds, the players decided simultaneously and concealed which share of this endowment they wanted to invest in maintaining the virtual dam. They wrote their decisions on reusable cards. The total of all individual contributions determined the group earning based on a non-linear pay-off function. In each round, the group earning was distributed equally among the players.

After playing five rounds with concealed decisions, we announced that the players' decision will be disclosed in the subsequent rounds. From round six onwards, contributions and earnings were written on a poster visible to all. After each round, players could openly discuss for five minutes.

After another five rounds, the facilitators chose one out of six game variations based on the content of the specific group's discussions. The game variation was played for five rounds.

We made incentivized payments in order to improve the reliability and validity of our games. All game decisions were entered into Excel-tables during the experiment sessions. A summary of the experiment design and the third-phase variations is given in Falk et al. [2]. The complete experiment protocol can be found under http://gamesforsustainability.org/India_village_dam_PG_game_ICRISAT_2016.pdf.
